# Discovery of melanin‐concentrating hormone receptor 1 in brown adipose tissue

**DOI:** 10.1111/nyas.14563

**Published:** 2021-01-27

**Authors:** Cécile Philippe, Eva‐Maria Klebermass, Theresa Balber, Oana C. Kulterer, Markus Zeilinger, Gerda Egger, Monika Dumanic, Carsten T. Herz, Florian W. Kiefer, Christian Scheuba, Thomas Scherer, Clemens Fürnsinn, Chrysoula Vraka, Katharina Pallitsch, Helmut Spreitzer, Wolfgang Wadsak, Helmut Viernstein, Marcus Hacker, Markus Mitterhauser

**Affiliations:** ^1^ Division of Nuclear Medicine Department of Biomedical Imaging and Image‐Guided Therapy Medical University of Vienna Vienna Austria; ^2^ Department of Pharmaceutical Technology and Biopharmaceutics University of Vienna Vienna Austria; ^3^ Ludwig Boltzmann Institute Applied Diagnostics Vienna Austria; ^4^ Division of Endocrinology and Metabolism, Department of Medicine III Medical University of Vienna Vienna Austria; ^5^ Faculty of Engineering University of Applied Sciences Wiener Neustadt Wiener Neustadt Austria; ^6^ Department of Pathology Medical University of Vienna Vienna Austria; ^7^ Division of General Surgery, Department of Surgery Medical University of Vienna Vienna Austria; ^8^ Institute of Organic Chemistry University of Vienna Vienna Austria; ^9^ Department of Pharmaceutical Chemistry University of Vienna Vienna Austria; ^10^ Center for Biomarker Research in Medicine – CBmed GmbH Graz Austria

**Keywords:** MCHR1, brown adipose tissue, PET, imaging, obesity

## Abstract

Although extensive research on brown adipose tissue (BAT) has stimulated optimism in the battle against obesity and diabetes, BAT physiology and organ crosstalk are not fully understood. Besides BAT, melanin‐concentrating hormone (MCH) and its receptor (MCHR1) play an important role in energy homeostasis. Because of the link between hypothalamic MCH neurons and sympathetic BAT activation via β‐adrenoceptors, we investigated the expression and physiological role of the MCHR1 in BAT. MCHR1 was detected in rodent and human BAT with RT‐qPCR and western blot analyses. *In vivo* imaging in rats used the glucose analog [^18^F]FDG and the MCHR1‐tracer [^11^C]SNAP‐7941. We found that the β3‐adrenoceptor (ADRB3) agonist CL316,243 increased [^11^C]SNAP‐7941 uptake in BAT. Additionally, a pharmacological concentration of SNAP‐7941—a low‐affinity ADRB3 ligand—stimulated [^18^F]FDG uptake, reflecting BAT activation. In cultured human adipocytes, CL316,243 induced *MCHR1* expression, further supporting a direct interaction between MCHR1 and ADRB3. These findings characterized MCHR1 expression in rodent and human BAT for the first time, including *in vitro* and *in vivo* data demonstrating a link between MCHR1 and the β3‐adrenergic system. The presence of MCHR1 in BAT emphasizes the role of BAT in energy homeostasis and may help uncover treatment approaches for obesity.

## Graphical abstract

Our findings here describe the expression of melanin‐concentrating hormone receptor 1 (MCHR1) in rodent and human brown adipose tissue (BAT). *In vitro* and *in vivo* data demonstrate a link between MCHR1 and the β3‐adrenergic system. The presence of MCHR1 in BAT emphasizes its role in energy homeostasis and may open new possibilities for treatment of obesity.

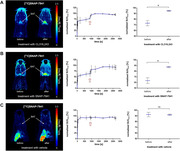

## Introduction

Since the rediscovery of active brown adipose tissue (BAT) in adult humans,^1^ BAT has become a topic of high interest in physiological and medical research. Through its ability to dissipate energy through heat generation, BAT has gained in significance as an antiobesity organ and may serve as a therapeutic target against obesity.^2^, ^3^, ^4^, ^5^ Besides cold exposure and food intake, other stimuli, including nutraceuticals and functional food, lead to an increase in norepinephrine that, among other things, activates β‐adrenoceptors (β1–β3) in BAT.^6^, ^7^ This signal triggers a physiological cascade in which triglycerides stored in white adipose tissue (WAT) are hydrolyzed to free fatty acids (FFAs) that, in turn, activate BAT‐specific uncoupling protein‐1 (UCP1) in the inner mitochondrial membrane. UCP1 activation promotes the generation of heat by uncoupling oxidative phosphorylation from ATP synthesis. This cascade can also be triggered by β3‐adrenoceptor (ADRB3) agonists such as CL316,243. Besides the combustion of released FFAs, other energy substrates, like glucose, are increasingly taken up from the blood by brown adipocytes during thermogenesis.^6^

While present in rodents throughout life, it was believed for a long time that BAT rapidly disappears in humans at a young age. This dogma was refuted in the last decade using a noninvasive imaging technique, positron emission tomography (PET).^1^, ^8^, ^9^, ^10^, ^11^The PET‐tracer 2‐[^18^F]fluoro‐2‐deoxy‐d‐glucose ([^18^F]FDG) is a surrogate for enhanced glucose utilization as observed in tumor metabolism and inflammation. Symmetrical [^18^F]FDG accumulation, which is highly atypical for tumors, was serendipitously observed in the neck and shoulder (supraclavicular) area of patients. Besides its main localization in the supraclavicular area, which corresponds to the interscapular BAT of rodents, it is also found in the mediastinum (paraaortic), as well as in the paravertebral and suprarenal regions.^1^, ^12^ Unlike WAT, BAT is metabolically favorable as an endogenous combustion system, and its abundance correlates with a leaner phenotype.^13^ Hence, the activation of BAT may represent an attractive strategy to treat obesity as well as type 2 diabetes (T2D), where active BAT might restore insulin sensitivity.^14^, ^15^

Another promising target for the treatment of obesity and T2D is melanin‐concentrating hormone receptor 1 (MCHR1).^16^, ^17^, ^18^, ^19^, ^20^, ^21^ This G protein–coupled receptor is predominantly expressed in the brain and involved in energy homeostasis.^22^, ^23^ Its natural ligand, melanin‐concentrating hormone (MCH),^24^ functions as a neuropeptide in mammals and is predominantly expressed in the lateral hypothalamus,^25^ one of the main brain regions involved in appetite regulation. Upregulation of MCH is present in obese mice and can be induced by fasting in naive mice.^26^ Furthermore, MCH‐overexpressing mice are hyperphagic, have elevated blood glucose levels, and develop excessive obesity.^27^ By contrast, MCH‐deficient mice are leaner than wild‐type mice due to hypophagia and a slight increase in energy expenditure.^28^ MCHR1 gene knockout mice further have a leaner physique, which results from hyperactivity, increased energy expenditure, and altered metabolism.^23^, ^29^

These findings stimulated the development of numerous MCHR1 antagonists as a possible treatment option for obesity. In several preclinical studies, the antagonists showed the expected effect of reducing food intake and increasing energy expenditure.^23^, ^30^ Moreover, Ito *et al*.^21^, ^31^ reported a trend toward enhanced FFA oxidation in isolated BAT after 1 month of MCHR1 antagonism. A link between BAT and the MCH system is further supported by neuronal MCH projections to brainstem regions harboring sympathetic neurons involved in BAT activation.^32^ As a result, PET‐tracers visualizing MCHR1 were developed^33^, ^34^, ^35^, ^36^, ^37^, ^38^, ^39^, ^40^, ^41^, ^42^ to enable its noninvasive *in vivo* quantification and monitoring of related pathologies, as well as to support drug development.

**Figure 1:**
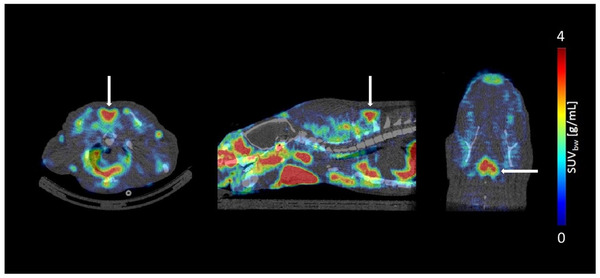
The μPET scan shows [^11^C]SNAP‐7941 uptake in rat BAT. Exemplary BAT uptake of [^11^C]SNAP‐7941 in rats shown with a transversal (left), sagittal (center), and coronal (right) μPET/CT summation image (0–45 minutes). White arrows indicate corresponding BAT uptake.

Taking advantage of the specific MCHR1 antagonist SNAP‐7941,^43^ we developed the radiolabeled analogue [^11^C]SNAP‐7941 and its fluoroethylated derivative [^18^F]FE@SNAP as specific PET‐tracers targeting the MCHR1 in the central nervous system.^33^, ^34^, ^35^, ^36^, ^37^, ^38^, ^39^, ^40^, ^41^ However, during a preclinical evaluation, both tracers were found to be P‐glycoprotein substrates.^35^, ^40^, ^44^ Unexpectedly, nonnegligible MCHR1‐tracer uptake in BAT of rats was repeatedly detected via small animal PET (μPET) (Fig. 1), pointing to the possibility of specific expression of MCHR1 in BAT. Because adrenergic receptors play an important role in BAT physiology, our next logical step was to exclude binding of the MCHR1 PET‐tracers to the predominant adrenergic receptor ADRB3;^4^ we demonstrated their affinities to ADRB3 to be in a micromolar range, which is irrelevant for PET imaging.^45^

Hence, the aim of our present study was to determine expression of MCHR1 in BAT, and to propose a more direct link between the MCH system and BAT physiology. Building on the discovery of this receptor in cultured murine brown adipocytes,^45^ we investigated and verified its expression in rodent and human BAT. Additionally, we used *in vivo* imaging to better understand MCHR1 expression in BAT and the implications of this expression.

## Materials and methods

### Animals

Adult male mice (BALB/cAnNRj and C57BL/6N) and rats (Sprague–Dawley HIM:OFA) were obtained from the Division of Laboratory Animal Science and Genetics, Himberg, Austria. The animals were kept under standard housing conditions (22 ± 1 °C; 40–70% relative humidity) on a constant 12‐h light/dark cycle with food and water supply *ad libitum*. All animals were treated according to the European Union rules on animal care, and respective experiments were approved by the Austrian Ministry of Science, Research and Economy (BMWF‐66.009/0029‐WF/V/3b/2015). In total, 36 HIM:OFA rats (24 for imaging and 12 for gene and protein analyses) and four mice (two BALB/cAnNRj and two C57BL/6N for gene and protein analyses) were used.

### Human adipose tissue samples

Adipose tissue samples were obtained from patients undergoing parathyroidectomy for primary hyperparathyroidism or elective thyroidectomy for benign goiter, Grave's disease, autonomous adenoma, or suspected thyroid carcinoma between March 2017 and April 2018. After providing informed written consent, all subjects who fulfilled the inclusion criteria (Supporting Information, File S1, online only) underwent thyroidectomy or parathyroidectomy at the Vienna General Hospital. This study has been approved by the Ethics Committee of the Medical University of Vienna (no. 2008/2014) and was conducted at the Department of Surgery, Division of General Surgery, and at the Division of Endocrinology and Metabolism, Department of Medicine III, Medical University of Vienna in accordance with the principles of the Declaration of Helsinki. Briefly, after the transverse skin incision in the anterior neck region, small parts of the subcutaneous fat (1–2 g) were removed. Afterward, during the preparation of the carotid artery for neuromonitoring of the nervus laryngeus recurrens (RLN, recurring laryngeal nerve), small specimens of the fat surrounding the carotid sheath were removed. During the further preparation of the RLN, deep neck fat surrounding the longus colli muscle was taken out. Finally, during the preparation of the caudal pole of the thyroid, small fat depots from the region were removed. In general, 0.5–2 g of the respective deep fat depot was excised.

### Isolation of human adipocyte precursor cells

Human adipocyte precursor cells (hAPCs) were isolated from fat specimens obtained during abdominoplasty from three different donors. Abdominal subcutaneous adipose tissue was excised from healthy female patients undergoing abdominoplastic surgery between June 2017 and May 2018. All subjects provided written informed consent. This study was approved by the Ethics Committee of the Medical University of Vienna and was conducted in accordance with the principles of the Declaration of Helsinki (EK 1149/2011 and EK 1032/2013). hAPCs were isolated and cultured as previously described.^46^ Confluent cells were induced (day 0) using a differentiation medium supplemented with 0.85 μM insulin (#I9278, Sigma‐Aldrich), 2 nM triiodothyronine (#I9278, Sigma‐Aldrich), 5 μM rosiglitazone (#R2408, Sigma‐Aldrich), 0.5 mM isobutylmethylxanthine (#I7018, Sigma‐Aldrich), and 1 μM dexamethasone (#D8893, Sigma‐Aldrich) for 2 days, followed by postdifferentiation medium supplemented with 0.85 μM insulin, 2 nM triiodothyronine, and 5 μM rosiglitazone. The postdifferentiation medium was changed every other day until day 6. Cells were stimulated with CL316,243 or vehicle (H_2_O) during the entire adipogenic differentiation.

### RNA extraction and reverse‐transcription quantitative PCR

The rodent tissue and human fat depot samples were snap‐frozen and stored at −80 °C before RNA isolation. For rodent *Mchr1* detection on the genome level, BAT of two BALB/cAnNRj and two C57BL/6N mice (6 and 8 weeks old, respectively), as well as of seven HIM:OFA rats (three animals aged ≤40 weeks and four aged 50, 90, 95, and 99 weeks) were analyzed. Two spleen and brain samples of BALB/cAnNRj mice and HIM:OFA rats served as the negative and positive controls, respectively. In the case of the four rats aged ≥50 weeks and all mouse tissues, organs were cut into halves to conserve a specimen for western blot (WB) analysis. All samples were ground using TRIzol® reagent (#15596026, Invitrogen). For rodent BAT samples, additional centrifugation steps at 4 °C were included in the extraction protocol in order to remove fat content. Subsequent to RNA extraction, RNA concentration and purity were checked with a NanoDrop device (Thermo Fisher Scientific). Reverse transcription was run with the qScript® cDNA Synthesis Kit (#95047, Quantabio), where after two‐step reverse‐transcription, quantitative PCR (RT‐qPCR) was performed with 2 μg DNA each, using the Luna® Universal qPCR Kit (#M3003, BioLabs) and the CFX96™ Real‐Time Detection System (Bio‐Rad).

In the case of human fat depot biopsies (*n = *6 for the carotid sheath, *n* = 19 for the thymus, *n* = 15 for the longus colli muscle, and *n = *18 for subcutaneous fat) and hAPCs (*n = *3), total RNA was also extracted using TRIzol (#15596026, Invitrogen), and samples were treated with DNase (#EN0521, Thermo Scientific) and reverse transcribed to cDNA using a High Capacity cDNA Reverse Transcription Kit with RNase inhibitor (#4374966, Applied Biosystems). RT‐qPCR was performed with 3 μg DNA each, using FastStart® Universal SYBR green (#04887352001, Roche) and a QuantStudio 6 RealTime PCR System (Applied Biosystems).

Relative mRNA expression of both rodent and human samples was determined using the ΔΔCt method, with β‐actin (rodent) or 36B4 (human) mRNAs as a normalization control. In the case of human tissue samples, only the deep cervical fat samples with significantly higher *UCP1* expression, compared with white fat depots (ΔCt > mean ΔCt WAT), were used for further analysis. Data were further processed with Excel 2013 (Microsoft® Office) and GraphPad Prism 6 (GraphPad Software Inc.).

All rodent and human primer sequences (eurofins) are described in the Supporting Information (online only).

### Protein extraction and WB

For MCHR1 detection at the protein level, the other halves of the murine samples and four rat BATs ≥50 weeks, previously analyzed with RT‐qPCR, were used. Additionally, two new brain and spleen samples and five fresh BAT samples from HIM:OFA rats aged ≤40 weeks were used. Tissues were extracted using an Ultra Turrax® tissue homogenizer (IKA, Germany) in the presence of a radioimmunoprecipitation assay (RIPA) buffer (#20‐188, Merck) and the recommended amount of standard protease inhibitor cocktail (#P2714, Merck). After centrifugation (14,500 × *g*, 4 °C), protein concentration in supernatants was determined with a bicinchoninic acid (BCA) kit (#PR 23227, Thermo Scientific). To avoid imprecise results caused by high fat content, BAT was centrifuged at least two to three times to remove the fatty layer. Gel electrophoresis was performed with 20 μg protein each on TGX™ precast gels (#456‐1083, Bio‐Rad), followed by semidry blotting and total protein staining (TPS) with Ponceau S (#P7170, Sigma‐Aldrich) to ensure consistent protein loading. Subsequently, nitrocellulose membranes (#10600004, GE Healthcare Life Science) were blocked with a 5% dry milk powder for 90 min at room temperature. Incubation with the primary antibody (rabbit polyclonal anti‐MCHR1 #PA5‐50705, RRID:AB_2636157, 1:1000, Thermo Fisher Scientific) was run at 4 °C, overnight, followed by thorough washing and a 1‐h incubation at room temperature with a goat anti‐rabbit IgG HRP conjugate (#A16104, RRID:AB_2534776, 1:2500, Thermo Fisher Scientific). Chemoluminescence imaging was performed with the Clarity™ Western ECL substrate detection kit (#170‐5060, Bio‐Rad) and the ChemiDoc™ Imaging System (Bio‐Rad).

Detected protein bands were normalized to TPS with Ponceau S (Fig. S1, online only), performing pixel analysis in the free image processing program ImageJ (the National Institutes of Health, Bethesda, MD). To this end, the pixel densities of the respective MCHR1 band were divided by those of corresponding TPS lanes, allowing for relative quantification. These data were further processed with Excel 2013 and GraphPad Prism 6.

### Chemistry

The MCHR1 PET‐tracer [^11^C]SNAP‐7941 was synthesized as previously described.^33^ [^18^F]FDG was prepared using a fully automated cassette‐based synthesizer (FASTlab, GE Healthcare) within the clinical routine production at the Vienna General Hospital, Austria. All PET‐tracers were physiologically formulated and fulfilled the quality control parameters. The corresponding unlabeled compound SNAP‐7941 ((±)‐methyl(4*S*)−3‐{[(3‐{4‐[3‐(acetylamino)phenyl]−1piperidinyl}propyl)amino]carbonyl}−4‐(3,4‐difluorophenyl)−6‐(methoxymethyl)−2‐oxo‐1,2,3,4‐tetra‐hydro‐5‐pyrimidenecarboxylate hydrochloride)) and the precursor SNAP‐acid ((±)−3‐{[(3‐{4‐[3‐(acetylamino)phenyl]−1‐piperidinyl}propyl)amino]carbonyl}−4‐(3,4‐difluorophenyl)−6‐(methoxymethyl)−2‐oxo‐1,2,3,4‐tetra‐hydro‐5‐pyrimidenecarboxylate acid)) were synthesized according to Schirmer *et al*.^47^ at the Departments of Pharmaceutical Chemistry and Organic Chemistry (University of Vienna, Austria). The ADRB3 agonist CL316,243 (#1499) was purchased from Tocris Bioscience.

### Small animal imaging

**Figure 2:**
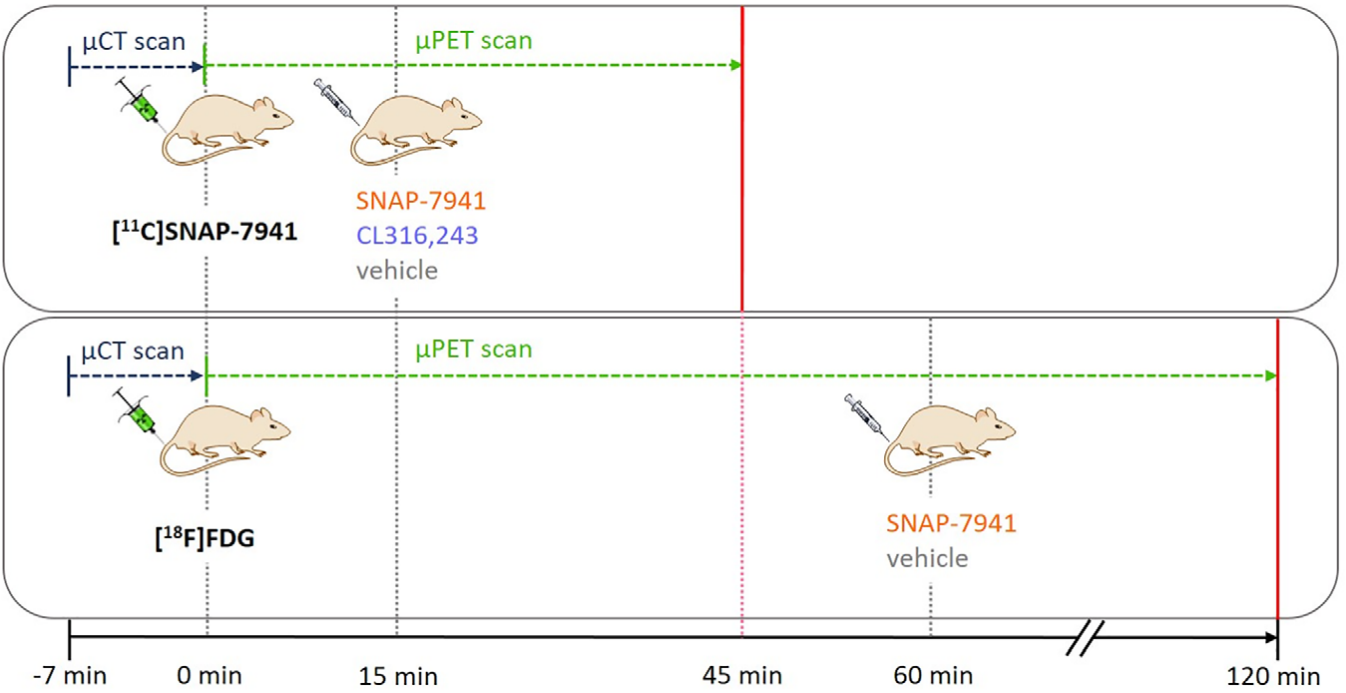
This figure represents the timeline of small animal imaging. After a μCT scan of 7 min, the respective tracer was applied and the μPET scan started. In the case of imaging with [^11^C]SNAP‐7941, SNAP‐7941, CL316,243, or vehicle was added 15 min after tracer application. The μPET scan was terminated after 45 minutes. As for the [^18^F]FDG scans, SNAP‐7941 or vehicle was added 60 min after tracer application and the μPET scan was stopped after 120 minutes.

Ten‐ to twelve‐week‐old male rats (397 ± 67 g) were anesthetized using 1.5–2% isoflurane with oxygen (1.5–2 L/min) and immobilized. Animals were warmed with a 37 °C positioning bed, except for imaging with [^18^F]FDG. Tracers (75.56 ± 5.48 MBq [^18^F]FDG (radiochemical purity >98%) or 82.7 ± 8.9 MBq [^11^C]SNAP‐7941 (molar activity: 44.33 ± 29.63 GBq/μmol; radiochemical purity >99%) as well as SNAP‐7941 (15 mg/kg body weight (BW)), CL316,243 (2 mg/kg BW), or the respective vehicles were injected into the lateral tail vein. The total volume applied did not exceed 1 milliliter. After a μCT scan of 7 min, dynamic PET imaging was acquired on a Siemens Inveon preclinical μPET/SPECT/CT system. Sixty minutes after [^18^F]FDG injection, SNAP‐7941 (*n = *4) or vehicle (*n = *4) was injected, and the PET scan was continued for another 60 minutes. For scans with [^11^C]SNAP‐7941, CL316,243 (*n = *6), SNAP‐7941 (*n = *6), or vehicle (*n = *4) was administered 15 min after tracer application, respectively. The total PET acquisition time was 45 minutes. Differences between [^18^F]FDG and [^11^C]SNAP‐7941 in PET acquisition time are due to the half‐lives of the respective nuclides (carbon‐11: 20.3 min; and fluorine‐18: 109.8 min) and the tracers’ equilibrium at 60 min ([^18^F]FDG) and 15 min ([^11^C]SNAP‐7941) after application. Figure 2 shows the timeline of small animal imaging.

### Image reconstruction and data postprocessing

The CT raw data were reconstructed with a Feldkamp algorithm using a Ramp filter followed by standard rat beam‐hardening correction and noise reduction (matrix size: 1024 × 1024; effective pixel size: 97.56 μm). The dedicated CT image data were calibrated to Hounsfield Units (HU). PET list mode data were sorted into three‐dimensional sinograms and reconstructed using an OSEM 3D/OP‐MAP scatter corrected reconstruction algorithm and a ramp filter (matrix size 128 × 128). The data were normalized and corrected for random, dead time, and radioactive decay. A calibration factor was applied to convert the activity information into absolute concentration units. Multimodal (μPET/CT) rigid‐body image registration and biomedical image quantification was performed using the image analysis software PMOD 3.8 (PMOD Technologies, Switzerland) and Inveon Research Workplace (IRW; Siemens Medical Solutions). Volumes of interest were outlined on multiple planes of the CT and PET images. Time activity curves (TACs) were calculated, normalized to dose and weight, and expressed as standardized uptake values (SUV; g/mL) to facilitate the comparison.

### *Ex vivo* metabolite analysis

After PET scanning with [^11^C]SNAP‐7941 and vehicle, anesthetized rats (*n = *4) were sacrificed by decapitation; BAT was harvested and subsequently homogenized with 250 μL 0.9% saline solution using an Ultra Turrax®. After the addition of acetonitrile (250 μL), the homogenate was vortexed and centrifuged (23,000 × *g*, 5 min, 4 °C) to remove fatty tissue and precipitated proteins. The recovery of the radioparent compound and potential radiometabolite extraction was measured using a gamma‐counter (2480 Wizard^2^, Perkin Elmer). The obtained supernatant was spiked with SNAP‐7941 and analyzed by analytical high‐performance liquid chromatography (HPLC) (mobile phase: (water:acetic acid = 97.5:2.5 %v/v; 2.5 g/L ammonium acetate; pH 3.5):acetonitrile 70:30 %v/v; stationary phase: Chromolith® Performance RP‐18e, 100–4.6 mm, #102129, Merck; flow: 1 mL/min; λ = 254 nm). The HPLC system (Agilent) comprised a diode array detector 1200 and a lead‐shielded BGO radiodetector (Elysia‐raytest GmbH, Germany). The ratio between the metabolite and intact tracer was decay corrected and calculated using quantitative HPLC analysis (GINAstar software, Elysia‐raytest GmbH, Germany).

### Statistical analysis

All experimental data are expressed as the mean ± SEM. Statistical testing was performed using GraphPad Prism 6. Differences among groups were proved using a two‐tailed parametric *t*‐test (paired in the case of small animal imaging data), and values of *P* < 0.05 were considered as statistically significant. Small animal imaging data were obtained using different batches of the radioligand. Multiple comparisons testing was performed using either ordinary one‐way ANOVA with Tukey's correction or ordinary two‐way ANOVA with Sidak's correction.

## Results

### Mchr1 mRNA is expressed in mouse, rat, and human BAT

**Figure 3:**
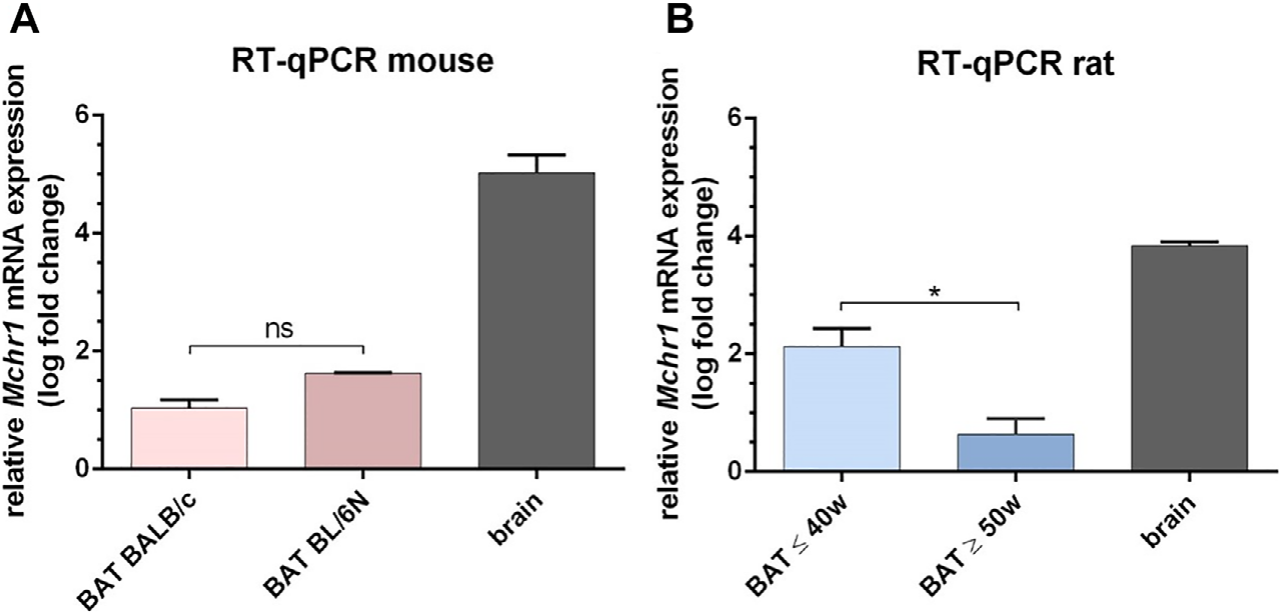
*Mchr1* mRNA is expressed in rodent BAT. Relative *Mchr1* expression in BALB/c brain and BAT of two different mouse strains (A, two animals each) and in the brain and BAT of HIM:OFA rats (B, three and four animals, respectively). Data are represented as the mean ± SEM from independent experiments. The Ct values were normalized to β‐actin, relative mRNA expression was calculated with respect to the negative control tissue spleen using the formula log(2^–ΔΔCt^). *Mchr1* expression on the genome level is significantly lower in rats aged ≥50 weeks (^*^ *P* < 0.05).

Referring to Kokkotou *et al*.,^48^ the brain and spleen were chosen as the positive and negative controls, respectively. *Mchr1* mRNA was quantified in the brain and spleen of BALB/c mice and BAT of two different strains of mice (Fig. 3A), as well as in the brain, spleen, and BAT of HIM:OFA rats (Fig. 3B). In both species, high relative *Mchr1* mRNA levels were found in the positive reference brain (mean log fold change 5.02 ± 0.29 (*n = *2) and 3.81 ± 0.06 (*n = *3), respectively). Lower levels were found in the BAT of rats (*n = *7) and all tested mice (*n = *4), with C57BL/6N having slightly higher levels (1.62 ± 0.01, *n = *2) than BALB/cAnNRj (1.07 ± 0.10, *n = *2) and rats showing a very heterogeneous expression dependent on their age (mean log fold‐change 2.12 ± 0.30 for ≤40 weeks old (*n = *3) and 0.63 ± 0.27 for ≥50 weeks old (*n = *4)). *Mchr1* mRNA was not detectable in the spleen of any of the tested mice and rats. Melting curve analysis confirmed the formation of a specific product corresponding to the positive control (Figs. S2 and S3, online only).

**Figure 4:**
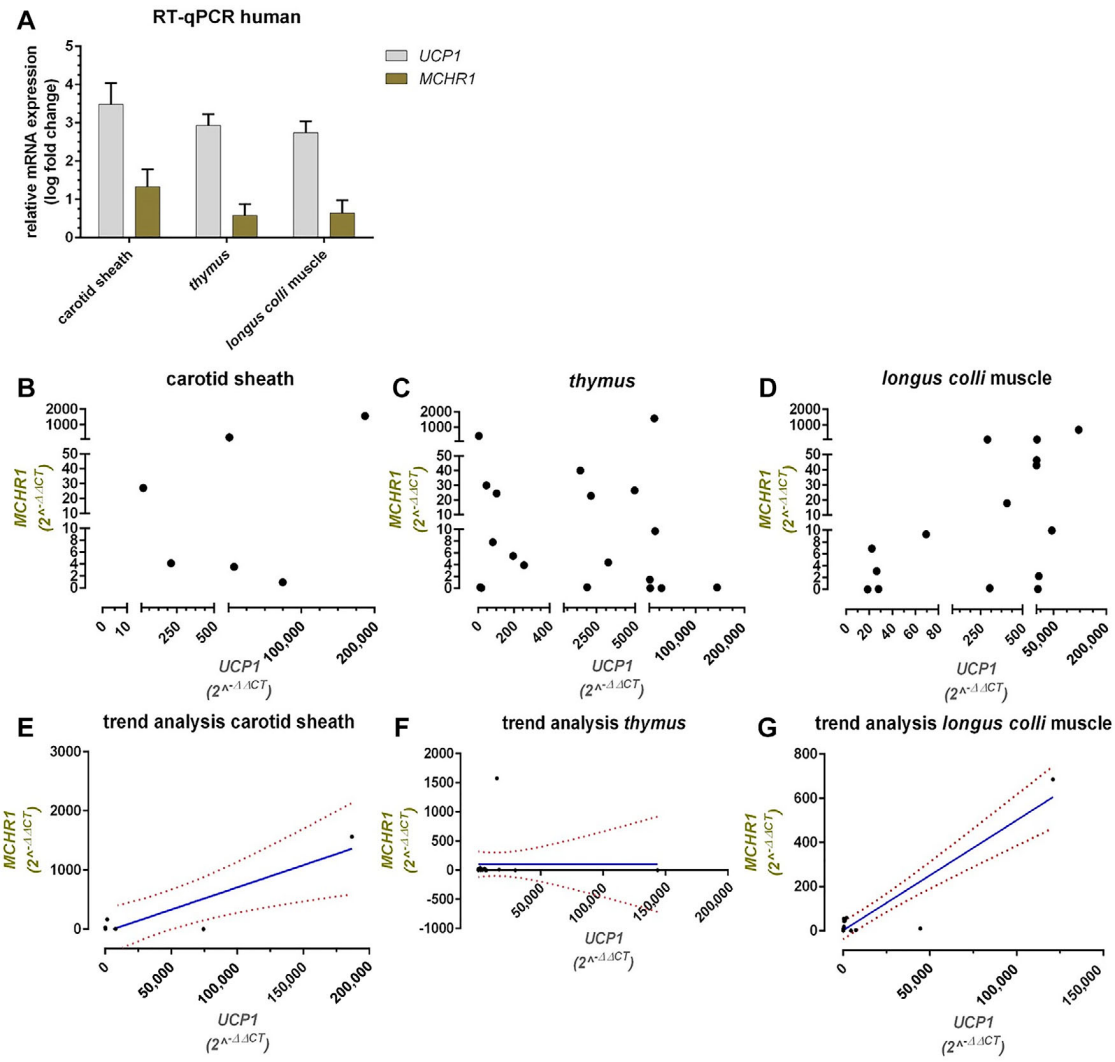
*MCHR1* mRNA is expressed in human BAT, but does not correlate with *UCP1* mRNA expression. Mean relative *MCHR1* and *UCP1* expression (±SEM) in human fat depot biopsies of the carotid sheath, thymus, and longus colli muscle (A). The Ct values were normalized to *36B4*, relative mRNA expression was calculated with respect to a subcutaneous WAT as the positive control with the formula log(2^–ΔΔCt^) (see Fig. S4 for the ΔCt values, online only). The respective 2^–ΔΔCt^ values for *MCHR1* were plotted against *UCP1* levels of the carotid sheath (B), thymus (C), and longus colli muscle (D) fat depots. Trend analyses (E–G) show a significant positive trend with regard to *MCHR1* versus *UCP1* for the carotid sheath (*R* ^2^ = 0.8196) and longus colli muscle samples (*R* ^2^ = 0.8529), but not for the thymus. Dotted red lines correspond to the confidence interval (95%). Only deep cervical fat samples with a significantly higher *UCP1* expression compared with white fat depots were used for analysis (*n = *6 for the carotid sheath, *n* = 19 for the thymus, and *n* = 15 for the longus colli muscle).

Receptor gene expression was further evaluated in several human biopsies of subcutaneous adipose tissue and fat depots surrounding the carotid sheath, thymus, and longus colli muscle (Fig. 4A). For the human samples, subcutaneous adipose tissue served as reference, known to express *MCHR1* ^49^ but low to no *UCP1*, as it mostly consists of white adipocytes. Generally, average ΔCt values (*MCHR1/B6B4*) of the thymus (12.14 ± 1.00, *n = *19) and longus colli muscle (11.94 ± 1.15, *n = *15) were in the range of the positive control (14.06 ± 0.82, *n = *18), but the mean ΔCt was significantly lower for carotid sheath (9.63 ± 1.63, *n = *6, *P* < 0.05, see Fig. S4, online only). Highest *MCHR1* levels were found in the carotid sheath samples (mean log fold change 1.33 ± 0.49, *n = *6) and lower ones in the thymus and longus colli muscle fat depots (0.58 ± 0.30 (*n = *19) and 0.64 ± 0.34 (*n = *15), respectively). Mean relative mRNA expression of *UCP1* of these samples was 3.48 ± 0.61 for the carotid sheath, 2.93 ± 0.29 for the thymus, and 2.74 ± 0.31 for the longus colli muscle, when compared with subcutaneous adipose tissue with very low *UCP1* levels. Detected *MCHR1* mRNA expression could not be correlated with *UCP1* mRNA levels, as a fit was not reasonable with given data points (Fig. 4B–D). However, trend analysis (Fig. 4E–G) revealed a significant positive trend for the carotid sheath (*R* ^2^ = 0.8196) and longus colli muscle samples (*R* ^2^ = 0.8529).

### MCHR1 is present in human adipocytes, and its expression is induced by chronic stimulation with CL316,243

**Figure 5:**
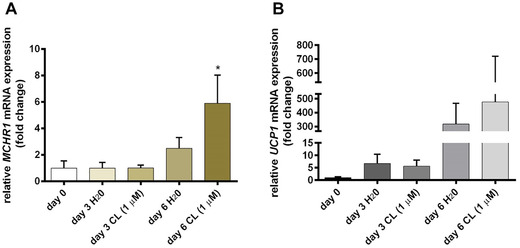
*UCP1* and *MCHR1* mRNA expression increases during differentiation of hAPCs and is induced by CL316,243. The mean relative *MCHR1* (A) and *UCP1* (B) mRNA expression (±SEM) on day 0 (before differentiation), day 3 (during differentiation), and day 6 (fully differentiated). *MCHR1* significantly (^*^ *P* < 0.05) increases with CL316,243 stimulation.

In order to study if MCHR1 is present on hAPCs and to investigate the influence of the ADRB3 agonist CL316,243 on MCHR1 gene expression, cells were isolated from three different donors. hAPCs were differentiated for 6 days using a standard adipocyte differentiation protocol. In order to obtain a BAT‐like phenotype, cells were stimulated with either control or CL316,243 (1 μM) during adipogenic differentiation. Cells were harvested before the start of differentiation (day 0), during differentiation (day 3), and when they were fully differentiated (day 6). Cell differentiation increased both UCP1 and MCHR1 gene expression over time (Fig. 5A and B). Interestingly, chronic stimulation with 1 μM CL316,243 determined a significant increase (*P* < 0.05) in MCHR1 gene expression on day 6 (Fig. 5A). These findings demonstrate that *MCHR1* mRNA is expressed in undifferentiated hAPCs as well as in fully *in vitro* differentiated adipocytes, and that its expression increases with CL316,243 stimulation.

### MCHR1 protein is expressed in rodent BAT

**Figure 6:**
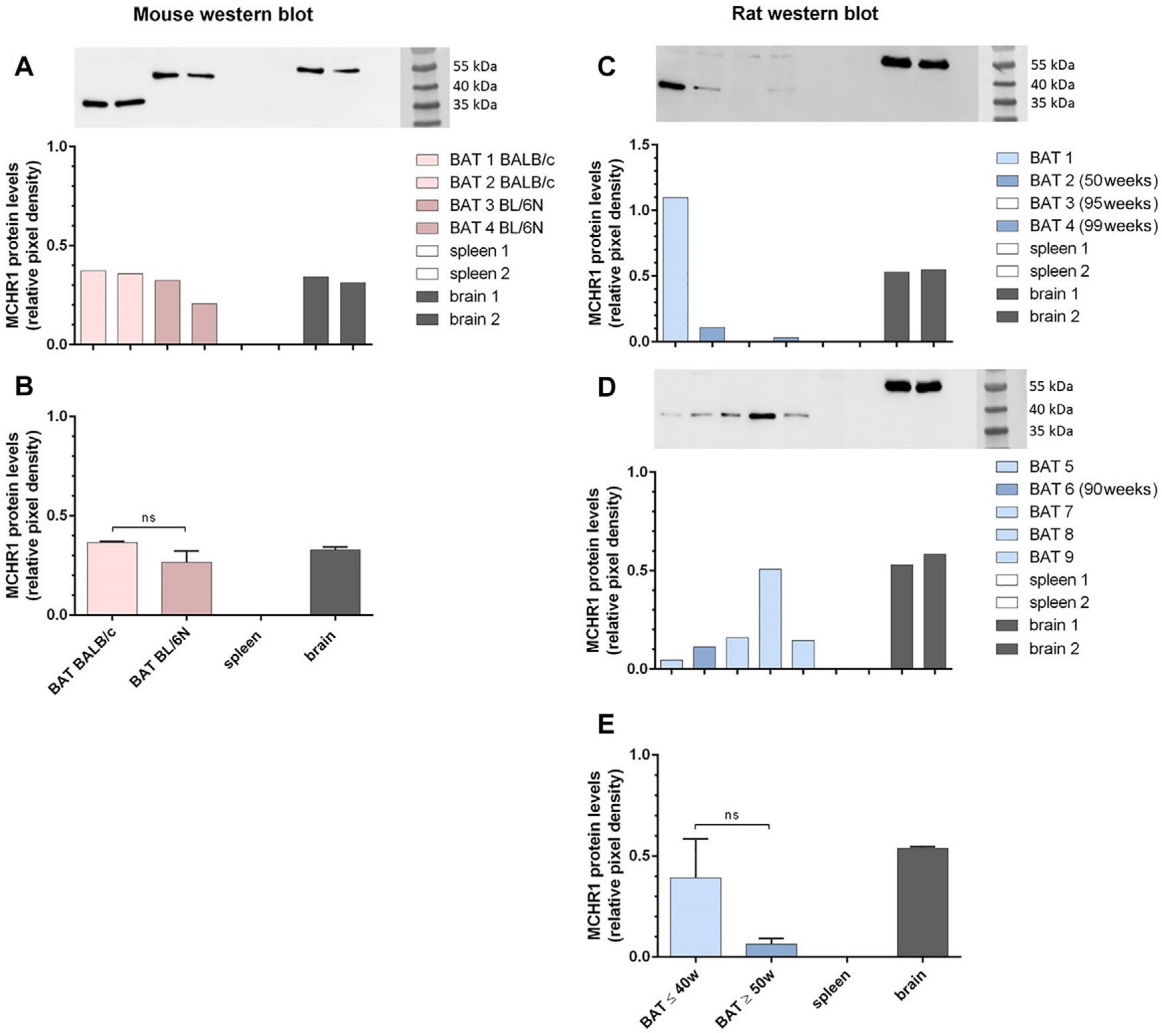
MCHR1 expression is comparable in two different mouse strains and dependent on age in HIM:OFA rats. MCHR1 expression in the brain and spleen of BALB/c mice and in BAT of two different mouse strains (A and B), as well as in the brain, spleen, and BAT of HIM:OFA rats of different age (C–E). (A, C, and D) The original western blots and matching relative pixel densities of the respective protein bands, normalized to Ponceau S (see Fig. S1, online only). In (C) and (D), all rats aged ≤40 weeks are shown in light blue and the ones aged ≥50 weeks in darker blue. Relative pixel densities of the protein bands of rat BAT probes in (C) were calculated for the 35–40‐kDa bands, not for the weaker ones visible at ∼55 kDa.

At the protein level, MCHR1 was discovered in all analyzed rodent brain and murine BAT samples (Fig. 6). Rats older than 50 weeks, in general, showed low (BAT 2, 4, and 6) or no MCHR1 protein (BAT 3) expression in BAT. However, MCHR1 was detected in all BAT samples of rats aged ≤40 weeks (Fig. 6C–E). Relative expression levels in BAT samples of younger rats were heterogeneous, with two samples showing a very high expression (BAT 1 and 8) comparable to or even exceeding expression MCHR1 levels found in the brain, and three samples (BAT 5, 7, and 9) had lower expression. All relative MCHR1 expression levels in both BALB/cAnNRj and C57BL/6N BAT were similar to those of the brain (Fig. 6A and B). In accordance with RT‐qPCR results, MCHR1 was not detected in the spleen. Generally, two bands (35–40 and ∼55 kDa) were detected in the tested BAT samples; in the brain, the MCHR1 band was always found at ∼55 kDa regardless of age, strain, and species. As for BAT, BL/6N mice exhibited an immunoreactive band at the same height, whereas BALB/c and HIM:OFA showed a lower molecular weight band. In some BAT samples from rats, we observed an additional weak band at ∼55 kDa.

### [^18^F]FDG uptake in BAT is increased by SNAP‐7941, while [^11^C]SNAP‐7941 uptake is enhanced by SNAP‐7941 and CL316,243

**Figure 7:**
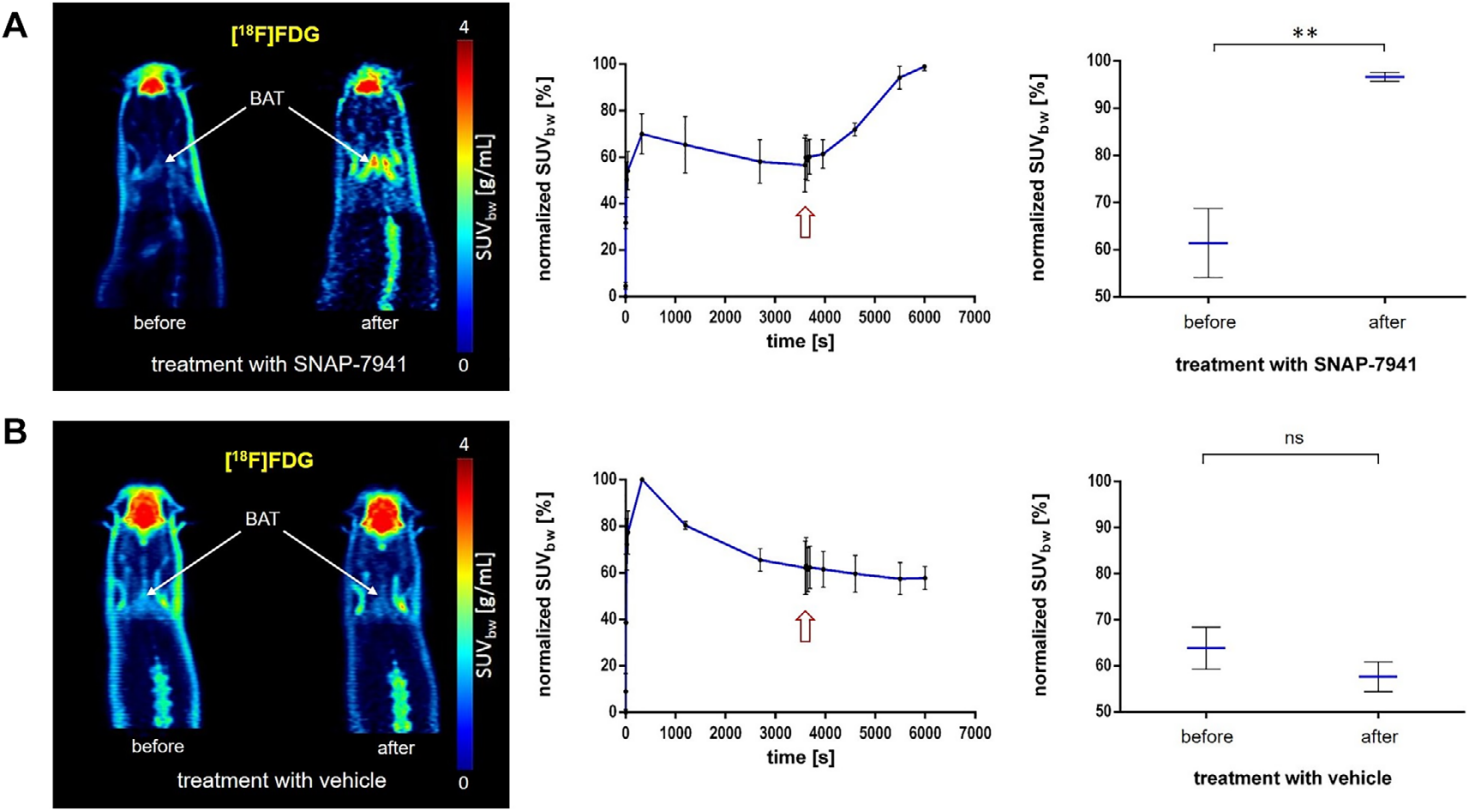
[^18^F]FDG uptake in BAT is significantly induced through SNAP‐7941. A representative BAT uptake of [^18^F]FDG shown in axial rat μPET summation images before and after treatment with 15 mg/kg SNAP‐7941 (A) and the dedicated vehicle (B), respectively. Corresponding TACs (center) and paired test statistics (right) are presented for both treatments with 15 mg/kg SNAP‐7941 (A) and the dedicated vehicle (B). Data are plotted as the mean ± SEM from independent experiments (*n = *4). Differences among groups were tested using a two‐tailed parametric paired *t*‐test (^**^ *P* ≤ 0.01). If not visible, error bars are within the margin of the symbols. Red arrows indicate the time point of SNAP‐7941 or vehicle addition.

Given that SNAP‐7941 binds to ADRB3 in higher concentrations, we aimed to study its effect on BAT activation with [^18^F]FDG imaging in rats. The administration of 15 mg/kg BW SNAP‐7941 (*n = *4) 60 min after [^18^F]FDG injection induced a significantly increased [^18^F]FDG uptake in BAT (*P = *0.0044, *df = *3, Fig. 7A). No effect was seen after addition of vehicle (*P* = 0.3277, *df = *3, *n = *4, Fig. 7B).

**Figure 8:**
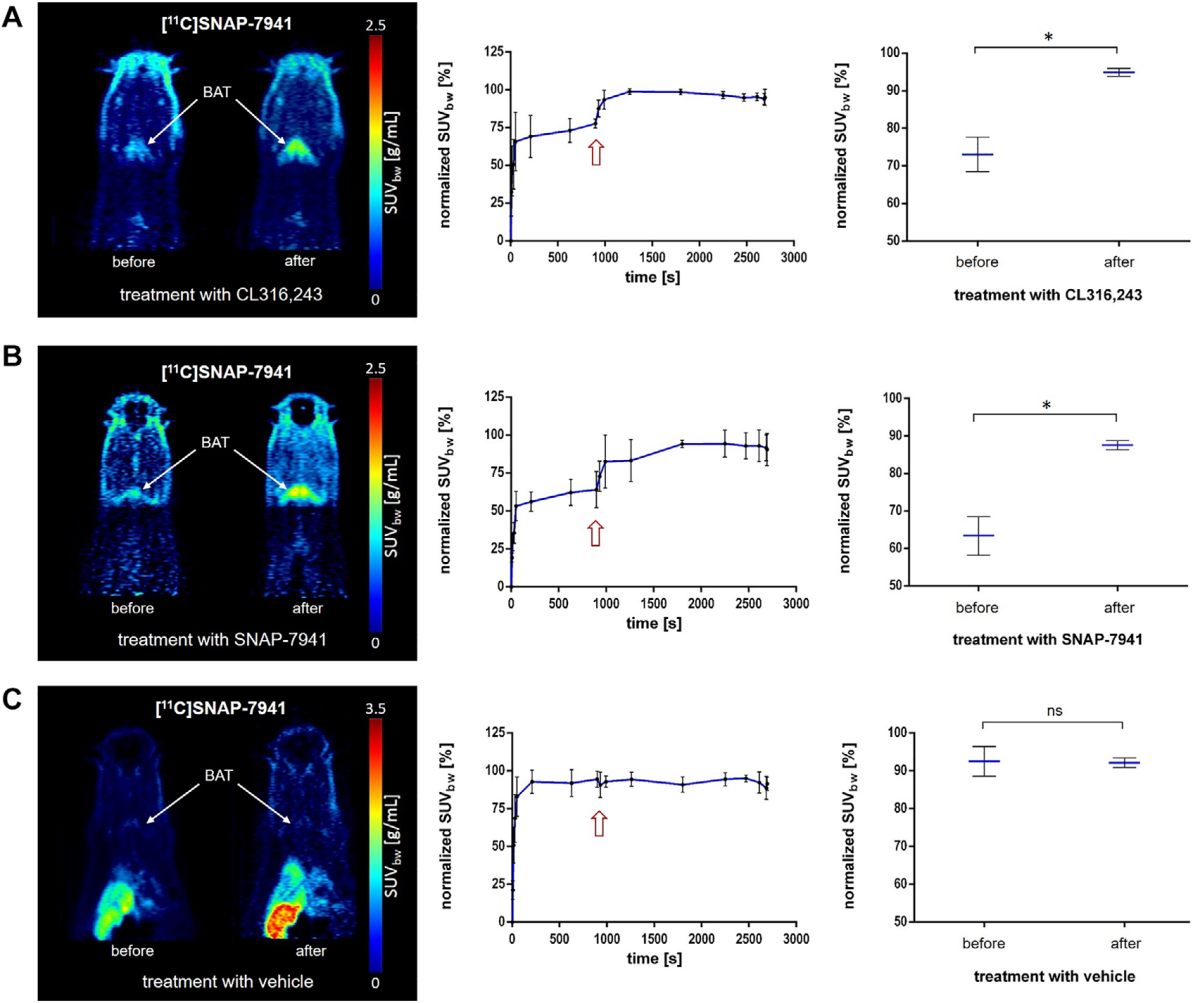
[^11^C]SNAP‐7941 uptake in BAT is significantly induced through SNAP‐7941 and CL316,243. Representative BAT uptake of [^11^C]SNAP‐7941 shown in axial rat μPET summation images before and after treatment with 2 mg/kg CL316,243 (A), 15 mg/kg SNAP‐7941 (B), and the dedicated vehicle (C). Corresponding TACs (center) and paired test statistics (right) are presented for the BAT uptake under treatment with 2 mg/kg CL316,243 (A), 15 mg/kg SNAP‐7941 (B), and the dedicated vehicle (C), respectively. Data are plotted as the mean ± SEM from independent experiments (*n = *4). Differences among groups were tested using a two‐tailed parametric paired *t*‐test (^*^ *P* ≤ 0.05). If not visible, error bars are within the margin of the symbols. Red arrows indicate the time point of SNAP‐7941, CL316,243, or vehicle addition.

Subsequently, in order to investigate MCHR1 *in vivo*, we performed imaging experiments with [^11^C]SNAP‐7941 using SNAP‐7941 for competition studies. Furthermore, as CL316,243 induced MCHR1 gene expression in hAPCs, we also investigated CL316,243 administration in μPET studies. The injection of 2 mg/kg BW CL316,243 (*n = *4, Fig. 8A), as well as 15 mg/kg (BW) SNAP‐7941 (*n = *4, Fig. 8B) 15 min after radiotracer application significantly enhanced BAT uptake in a similar range (*P* = 0.0287, *df = *2). Again, the administration of vehicle (*n = *4) had no influence on [^11^C]SNAP‐7941 BAT uptake (*P* = 0.9469, *df = *3, Fig. 8C). Four rats, however, had a high initial uptake of [^11^C]SNAP‐7941 (SUV > 2.5) and were excluded. In those rats, the administration of SNAP‐7941 (*n = *2) or CL316,243 (*n = *2) after 15 min caused either displacement or had no effect (data not shown).

### [^11^C]SNAP‐7941 is metabolically stable in BAT

In order to ensure the detected PET signal resulted from intact radiotracer, we determined potential radiometabolites of [^11^C]SNAP‐7941 in BAT using HPLC. Two hydrophilic radiometabolites at 2‐ and 4‐min retention time occasionally appeared in very limited quantities. The intact [^11^C]SNAP‐7941 (*n = *4; 94.0 ± 5.2%) was still present 45 min after tracer application. The recovery of the radio‐parent compound and radiometabolites after tissue preparation was >90%. A representative chromatogram is shown in the Supporting Information (Fig. S5, online only). This proves the stability of [^11^C]SNAP‐7941 during the scan period.

## Discussion

Collectively, our results prove presence of MCHR1 in rodent and human BAT and highlight [^11^C]SNAP‐7941 as a potential alternative PET‐tracer for BAT imaging. Since serendipitous observations with PET led to the rediscovery of BAT in adult humans, it may be seen as a coincidence that the same technique finally led to the detection of MCHR1 in BAT.

After we unexpectedly observed MCHR1‐tracer uptake in this tissue, we investigated an eventual binding to the ADRB3, the receptor predominantly involved in BAT activation. The sequence similarity between β‐adrenoceptors and MCHR1 already suggests the off‐target effects of MCHR1 antagonists.^50^ Both MCHR1 antagonists, SNAP‐7941 and FE@SNAP, were shown to bind to the ADRB3 only in a micromolar range (SNAP‐7941: *K_i_*_ = _14.5 ± 0.3 μM, FE@SNAP: *K_i_* = 65.1 ± 2.9 μM),^45^ demonstrating a high selectivity against the target protein. As PET‐tracers are applied in a pico‐ to nanomolar range, PET imaging with [^11^C]SNAP‐7941 only depicts MCHR1 binding.

Since we had established the superiority of [^11^C]SNAP‐7941 in terms of metabolic stability, affinity, and selectivity in a previous study,^40^ we chose the carbon‐11 tracer to further investigate the *in vitro* uptake in murine brown adipocytes.^45^ In uptake experiments with immortalized and differentiated murine brown adipocytes, SNAP‐7941 led to an ∼23% blocking of [^11^C]SNAP‐7941 uptake, whereas ADRB3 agonist CL316,243 had a significantly lower effect. Hence, these data suggested that MCHR1 is expressed in these cultured murine brown adipocytes.

In order to put these findings into a more physiological context, we examined MCHR1 expression in rodent and human BAT. To also take strain differences into account, we analyzed BL/6N as well as BALB/c mice, and found that BL/6N had slightly higher levels of *Mchr1* mRNA than BALB/c. As for HIM:OFA rats, *Mchr1* mRNA in BAT was generally detected to a greater extent than in mice (Fig. 3A), although significantly lower gene expression correlated with increasing age (Fig. 3B). BAT function is known to decrease with age not only in humans but also in rodents. It has been reported that substantial morphological remodeling of BAT takes place in 6‐month‐old mice, resulting in the formation of hypertrophied brown adipocytes with larger lipid droplets.^51^ Consequently, one could suggest a transformation of BAT as a combustion system toward becoming an energy‐storing organ. This might explain why older animals are more susceptible to obesity. Owing to this age‐related morphological change, *Mchr1* mRNA expression could be affected as well.

Despite the comparably low gene expression of BAT, we detected relatively high MCHR1 protein levels comparable to, or exceeding, those in the brain in some cases (6 out of 13 BAT samples, Fig. 6). This might be due to either high mRNA turnover or high MCHR1 protein stability. Furthermore, MCHR1 gene expression was in a similar range in both strains of mice aged 6–8 weeks, despite different mRNA levels (Fig. 6A and B). MCHR1 was found to be heterogeneously expressed in rats of various ages (Fig. 6C–E). The two different immunoreactive bands (35–40 kDa and ∼55 kDa) detected in BAT samples may be explained by alternate splicing variants of the pre‐RNA as described for human *MCHR1*.^52^ More importantly, there is also substantial posttranslational modification of the protein. Saito *et al*.^53^ previously reported posttranslational N‐terminal glycosylation of MCHR1, resulting in bands between 35 and 65 kDa molecular weight. Depending on the glycosylation pattern, the uppermost or lower bands can be absent. Because of tissue differences, loading controls based on housekeeping genes were found to be inappropriate; therefore, normalization to Ponceau S was performed (Fig. S1, online only).

In human samples, *MCHR1* and *UCP1*, an indicator for BAT, were divergently expressed among the tested samples (Fig. 4A–D). Owing to this heterogeneity, as well as the fact that most data points represent either comparatively low or very high *UCP1* expression, the fit we performed using Pearson's correlation was distorted and, therefore, not deemed valid (data not shown). Nevertheless, trend analysis confirmed a positive correlation of *UCP1* and *MCHR1* expression in the carotid sheath and longus colli muscle fat depots (Fig. 4E–G). However, as the expression of *UCP1* alone is not representative of BAT activity,^54^ the correlation between *UCP1* and *MCHR1* might not be relevant. Antonacci *et al*.^55^ measured BAT thermogenesis by hyperpolarized ^129^Xe MR thermometry after adrenergic stimulation of *UCP1*‐KO mice and showed significant, UCP1‐independent activation of thermogenesis. In addition, Long *et al*.^56^ described another protein (PM20D1) as a mitochondrial uncoupler protein distinct from UCP1 in BAT, again indicating that thermogenesis in BAT is more complex than anticipated.

Since we verified the expression of the receptor in both rodent and human BAT and had a promising MCHR1 PET‐tracer at hand, we performed preclinical *in vivo* imaging studies to better understand the physiological role of MCHR1 in BAT as well as to visualize the activating effect of MCHR1 antagonists. To investigate the latter, the most used PET‐tracer for BAT imaging, [^18^F]FDG^57^ was applied. Interestingly, we observed an enhanced [^18^F]FDG uptake in BAT after administration of SNAP‐7941 (15 mg/kg (BW)) in rats (Fig. 7A). [^18^F]FDG uptake in BAT as a measure for BAT activity is also enhanced by the ADRB3 agonist CL316,243, as reported by Mirbolooki *et al*.^58^ Consequently, one could assume that both ADRB3 agonism and MCHR1 antagonism have the same activating effect on BAT. The mechanism of BAT activation through MCHR1 antagonists has been previously postulated by Ito *et al*.^31^ However, since SNAP‐7941 also binds to the ADRB3 when administered in a high concentration,^45^ we cannot exclude that the observed BAT activation is at least partially attributed to ADRB3 agonism.

As the uptake of glucose in BAT is strongly enhanced during thermogenesis, activation of BAT is a prerequisite for [^18^F]FDG uptake. BAT mass and activation can be modulated by various factors, such as sex, age, and body mass. Most importantly, BAT is stimulated by low temperatures.^13^ Thus, in preclinical studies, which investigate BAT with [^18^F]FDG, rodents are usually cooled down to 4–8 °C for several hours before the μPET scan to enable [^18^F]FDG uptake in BAT. As we did not aim to study cold‐induced BAT activation, animals were not cooled down prior to the [^18^F]FDG scan. However, in order to provoke enough [^18^F]FDG uptake to study the effects of SNAP‐7941 addition, animals were not actively warmed during the dynamic μPET scan.

Regarding the experiments with [^11^C]SNAP‐7941, we observed tracer uptake in BAT of rats warmed with a 37 °C positioning bed during the scan. In contrast to [^18^F]FDG, the uptake of [^11^C]SNAP‐7941 is based on specific receptor binding and does not reflect glucose turnover. Moreover, *ex vivo* metabolite analyses demonstrated the stability of [^11^C]SNAP‐7941 in BAT, therefore, imaging results are not affected by radiometabolites.

Even though the previously mentioned *in vitro* preblocking experiments showed a significant reduction in [^11^C]SNAP‐7941 uptake by SNAP‐7941, *in vitro* competition experiments showed only marginal displacement (unpublished data). Nevertheless, we decided to conduct competition experiments to determine specific binding *in vivo* in order to observe effects within the same individual, thereby avoiding any influence of biodiversity. Paradoxically, in these studies, SNAP‐7941 did not decrease [^11^C]SNAP‐7941 uptake in BAT, as one would anticipate, but led to a binding enhancement (Fig. 8B). Furthermore, an enhancement of [^11^C]SNAP‐7941 uptake was not detected if the basal uptake in BAT was already high (initial SUV >2.5). The same pattern of [^11^C]SNAP‐7941 uptake in BAT was observed after the administration of CL316,243 (Fig. 8A). ADRB3 agonism is known to increase blood flow, and BAT is highly vascularized. However, the plateau of *in vivo* [^11^C]SNAP‐7941 uptake after SNAP‐7941 or CL316,243 addition (Fig. 8A and B) is more likely to reflect specific binding as there are no redistribution effects. In the case of high basal uptake, the addition of nonradioactive SNAP‐7941 led to either slight displacement or had no effect (data not shown). When comparing those animals’ age, weight, time of measurement, molar activity of the tracer, and temperature of the positioning bed, no apparent differences were observed. However, we observed heterogeneous MCHR1 expression in BAT among the tested rats (Fig. 6C–E). Such diverging expression levels could explain a varying initial [^11^C]SNAP‐7941 uptake. Additionally, even though the *in vivo* measurements were following a standardized protocol, we cannot exclude that the animals were in different physiological conditions causing these diverging effects. Regarding the shape of TACs, an enhancement of both [^18^F]FDG and [^11^C]SNAP‐7941 uptake after SNAP‐7941/CL316,243 addition is clearly visible at a first glance. Yet, if one looks closer, SNAP‐7941 causes a comparatively slow and continuous increase of [^18^F]FDG uptake in all studied rats, which probably reflects BAT activation triggered by SNAP‐7941 binding to the ADRB3. In the case of [^11^C]SNAP‐7941, however, SNAP‐7941/CL316,243 addition causes a sharp increase in BAT uptake that is rapidly reaching a plateau and can only be observed in animals with low basal uptake. The difference in TACs could be explained by [^18^F]FDG uptake reflecting metabolism (BAT activation) and [^11^C]SNAP‐7941 uptake depicting receptor binding. One cannot exclude that the enhancement of [^11^C]SNAP‐7941 BAT uptake after SNAP‐7941 or CL316,243 addition might root in some coupling between the ADRB3 and MCHR1, given that SNAP‐7941 binds to the ADRB3 in higher concentrations and has the same enhancing effect as CL316,243, a known ADRB3 agonist. This supposed link could be supported by our RT‐qPCR data of cultured human precursor adipocytes, where chronic treatment with CL316,243 significantly increases *MCHR1* mRNA expression (Fig. 5A). Although this strongly suggests a connection between BAT activation and MCHR1 expression, the observed mechanism cannot explain the prompt increase in [^11^C]SNAP‐7941 uptake in BAT through ADRB3 agonism since transcriptional effects can be excluded in this short time frame. However, MCHR1 is not only localized in the cell membranes; also MCHR1‐containing vesicles have been reported after receptor internalization.^59^, ^60^ The rapid nature of the observed effects of SNAP‐7941 and CL316,243 on [^11^C]SNAP‐7941 BAT uptake could, therefore, be explained by MCHR1 resurfacing. Furthermore, neither the exact binding mechanism of SNAP‐7941 nor an interaction of SNAP‐7941 and CL316,243 with other BAT‐specific receptors is known. Additionally, preliminary competition experiments with a structurally different MCHR1 antagonist GW‐803430 showed similar effects on [^11^C]SNAP‐7941 uptake in rat BAT (unpublished data), proposing a general link between MCHR1 antagonists and the β‐adrenergic system. BAT physiology is influenced by a plethora of factors, including volatile anesthetics like isoflurane^61^ applied in this study, and the identification of the underlying mechanism provoking these effects remains an open challenge.

In conclusion, the previously presumed MCHR1 expression in BAT was more directly demonstrated in rodents and humans. This points to a direct action of MCH in BAT and emphasizes the physiological role of BAT as an endocrine organ. Although MCHR1 is present in BAT, we do not yet fully understand its involvement in BAT physiology and its effect on the organism's metabolic network. That, as well as the link between MCHR1 and the metabolically relevant adrenergic receptors in BAT, should be subject to further investigations. In the era of precision and personalized medicine, whole‐body PET can help unwire the physiological connections of BAT and organ crosstalks. To our knowledge, [^18^F]FDG can fail as a surrogate of BAT thermogenesis and is, as a tracer reflecting glucose metabolism in general, highly unspecific. Therefore, an alternative PET‐tracer for BAT imaging is of interest. As BAT has become a pharmacological target to treat metabolic diseases, the detection of MCHR1 in BAT may be another component that could help to curb the obesity pandemic.

## Author contributions

C.P., E.M.K., and M.M. conceived the study and designed the experiments. C.P., E.M.K., T.B., O.C.K., M.D., C.V., and M.Z. performed the experiments. C.P. performed the radiosyntheses of [^11^C]SNAP‐7941. G.E. designed the primers for RT‐qPCR, helped with the establishment of the protocol for RT‐qPCR, and interpretation of the data. C.T.H. and C.S. provided the human fat depot samples. K.P. and H.S. synthesized the precursor compound for radiolabeling and standard compound. M.Z. did the data postprocessing of the μPET/CT scans and statistical analysis. C.P., E.M.K., and O.C.K. analyzed the data and composed the draft. M.M., G.E., W.W., F.W.K., C.F., T.S., M.H., and H.V. helped with outcome interpretation and reviewed the manuscript. All authors have given approval to the final version of the manuscript.

## Competing interests

The authors declare no competing interests.

## Supporting information


**Figure S1**. Total protein staining with Ponceau S corresponding to western blots depicted in Figure 6.

**Figure S2**. Example of rat RT‐qPCR melting curves.

**Figure S3**. Example of mouse RT‐qPCR melting curves.

**Figure S4**. The ΔCt values (*MCHR1‐36B4*) for human subcutaneous fat probes (*n = *18) and probes of adipose tissue surrounding the carotid sheath (*n = *6), thymus (*n = *19), and longus colli muscle (*n = *15).

**Figure S5**. A representative HPLC chromatogram of rat BAT 45 min after [^11^C]SNAP‐7941 application (top: UV channel, bottom: radioactivity channel). The analyzed sample was spiked with the reference compound SNAP‐7941.

**Supplementary Material File S1**.
