# Supplementary figures and images for: Discovery of melanin‐concentrating hormone receptor 1 in brown adipose tissue
Source: Ann N Y Acad Sci. 2021 Jan 27;1494(1):70–86. doi: 10.1111/nyas.14563 (PMC8248337; doi:10.1111/nyas.14563)

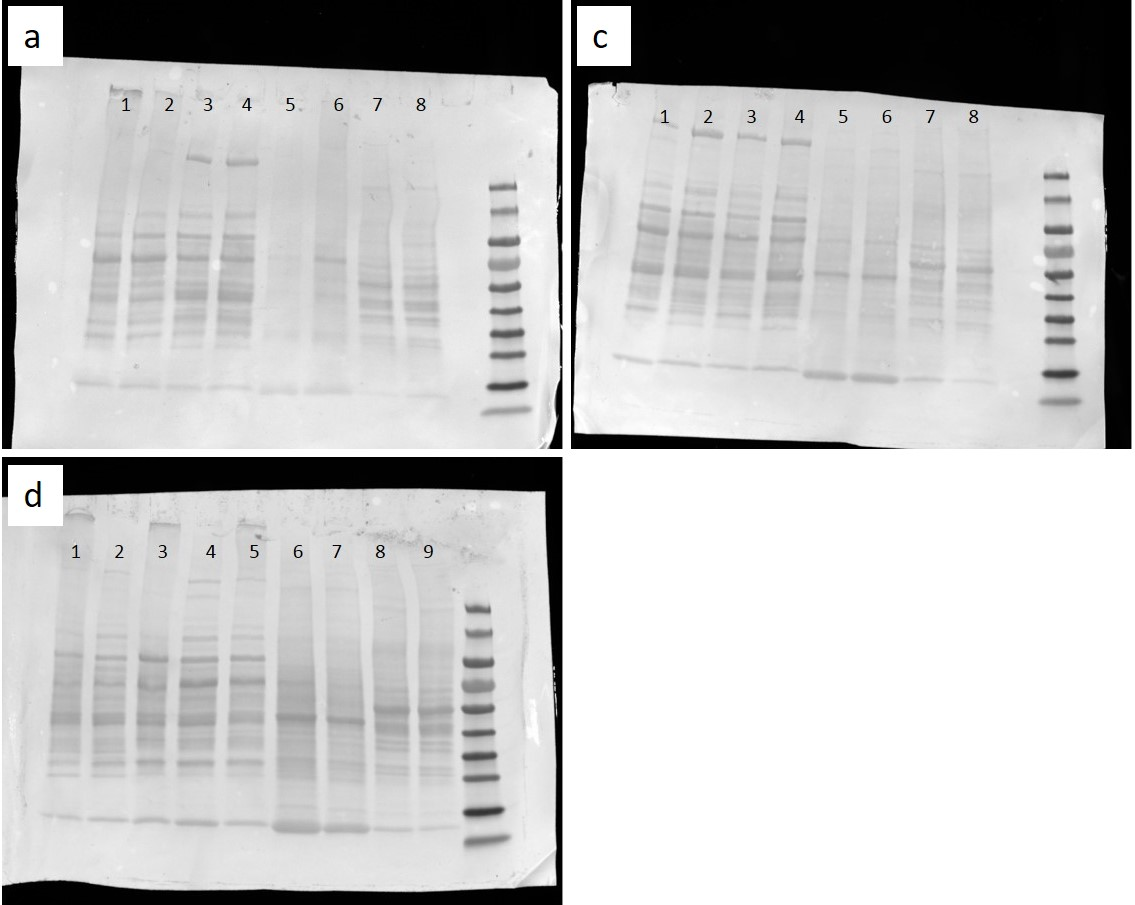

Supplement: Supplementary file 1 — Figure S1. Total protein staining with Ponceau S corresponding to western blots depicted in Figure 6. [file NYAS-1494-70-s002.tif]

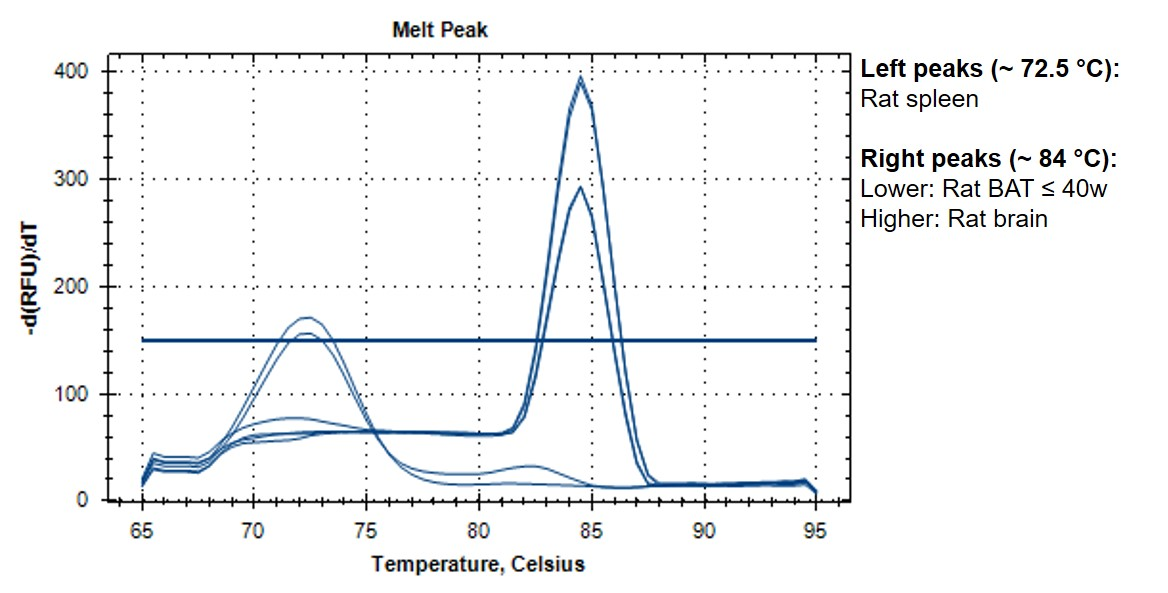

Supplement: Supplementary file 2 — Figure S2. Example of rat RT‐qPCR melting curves. [file NYAS-1494-70-s006.tif]

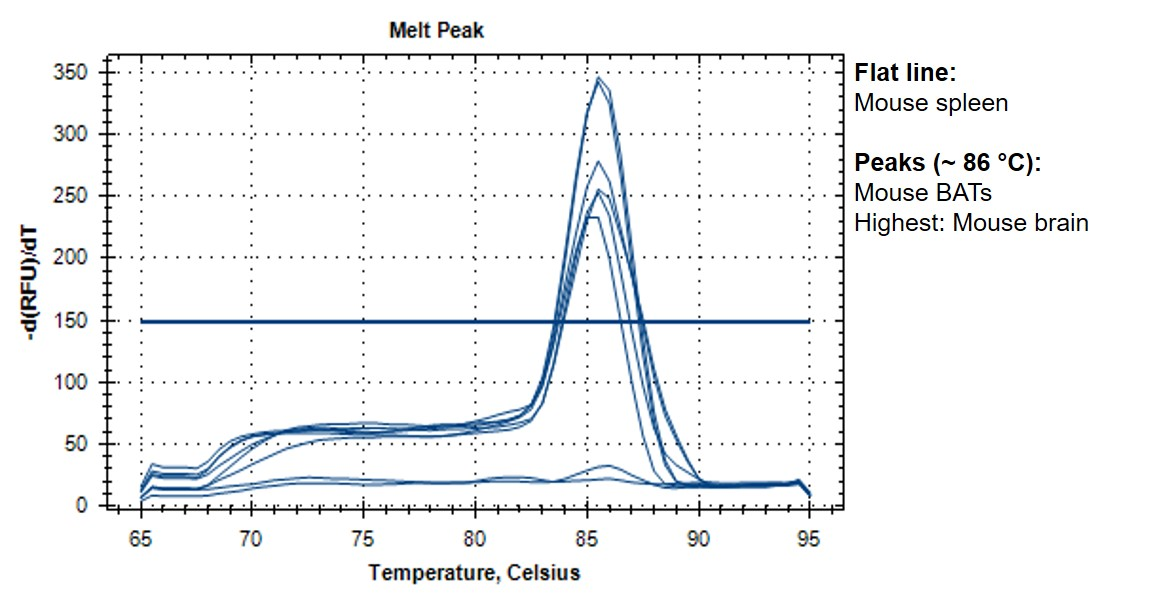

Supplement: Supplementary file 3 — Figure S3. Example of mouse RT‐qPCR melting curves. [file NYAS-1494-70-s001.tif]

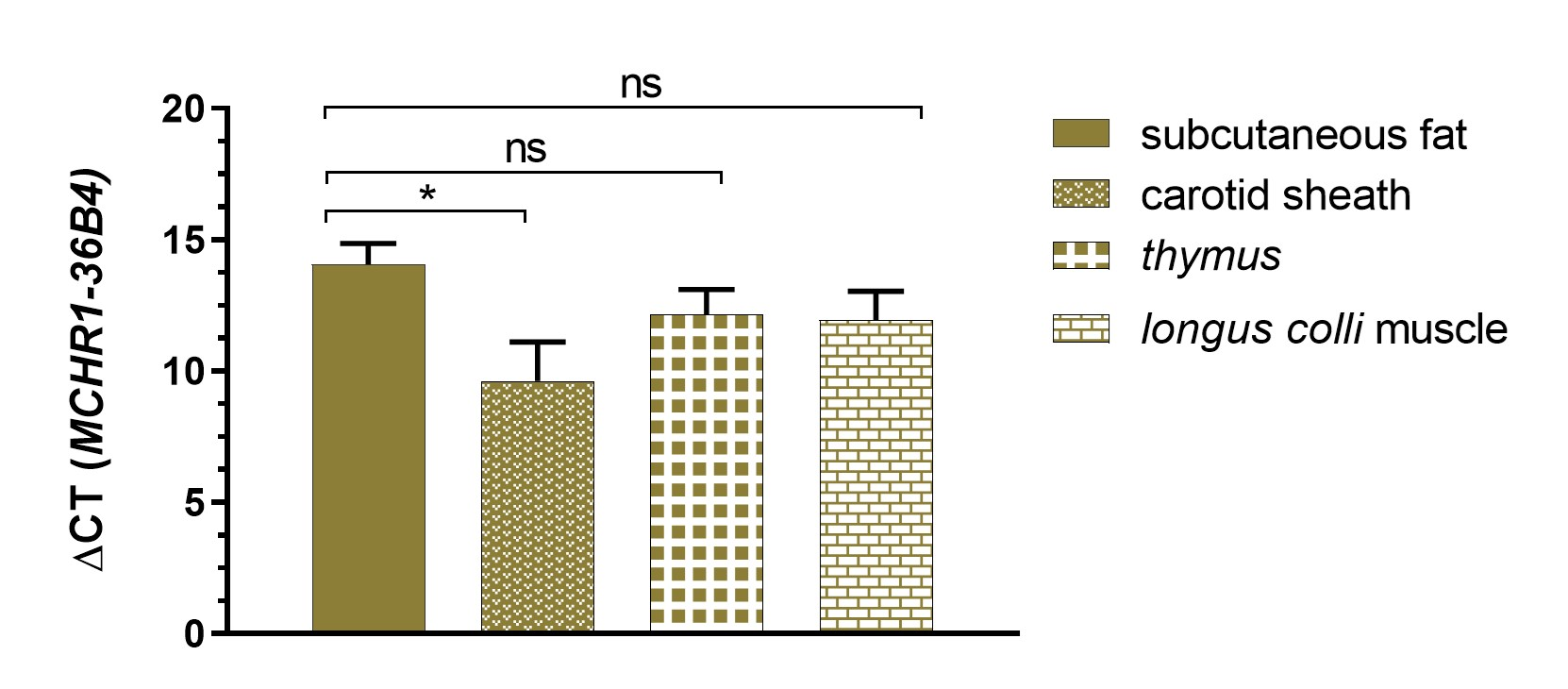

Supplement: Supplementary file 4 — Figure S4. The ΔCt values (MCHR1‐36B4) for human subcutaneous fat probes (n = 18) and probes of adipose tissue surrounding the carotid sheath (n = 6), thymus (n = 19), and longus colli muscle (n = 15). [file NYAS-1494-70-s004.tif]

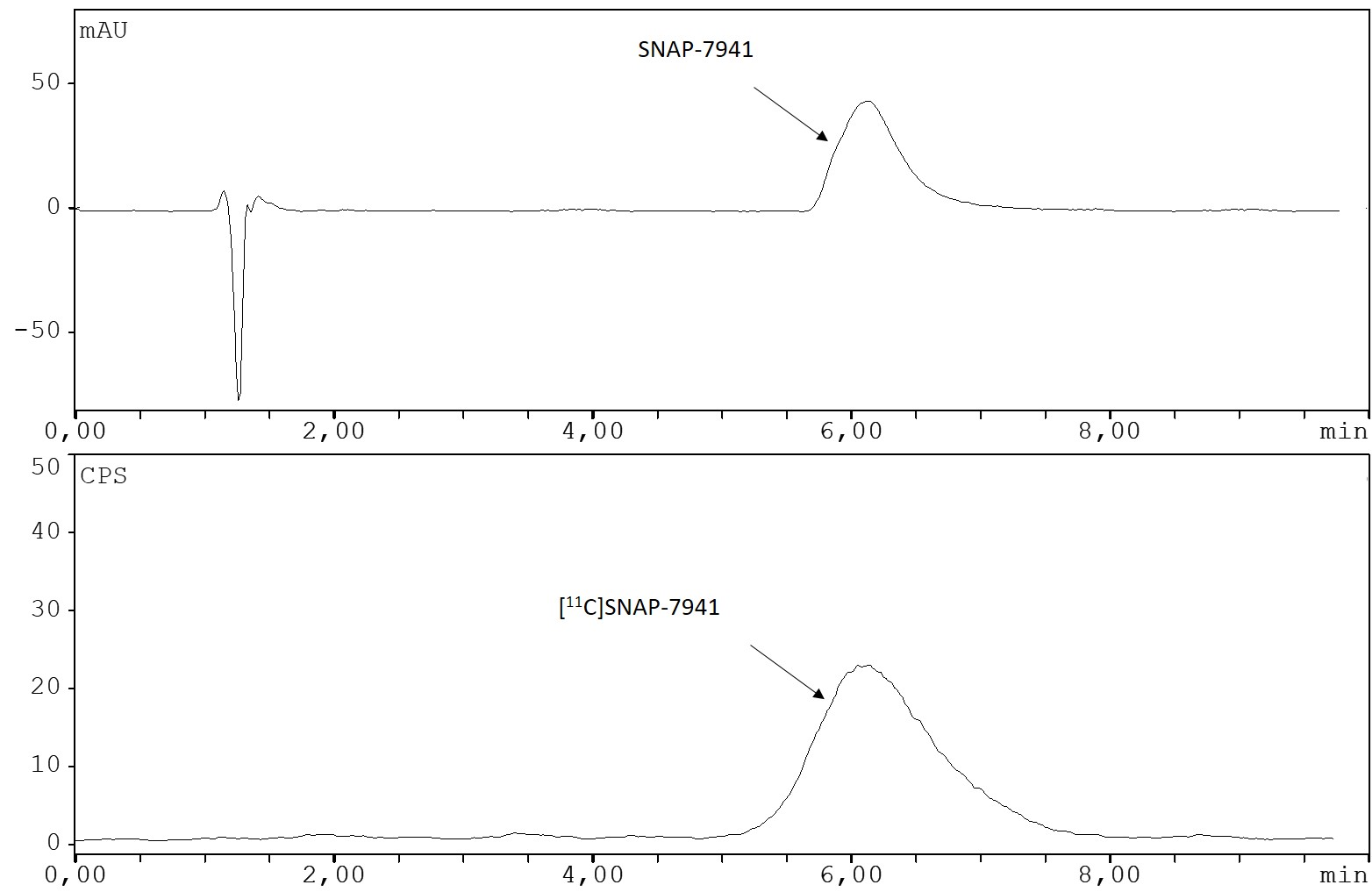

Supplement: Supplementary file 5 — Figure S5. A representative HPLC chromatogram of rat BAT 45 min after [11C]SNAP‐7941 application (top: UV channel, bottom: radioactivity channel). The analyzed sample was spiked with the reference compound SNAP‐7941. [file NYAS-1494-70-s003.tif]
