# Supplementary material for: Discovery of melanin‐concentrating hormone receptor 1 in brown adipose tissue
Source: Ann N Y Acad Sci. 2021 Jan 27;1494(1):70–86. doi: 10.1111/nyas.14563 (PMC8248337; doi:10.1111/nyas.14563)
Supplement: Supplementary file 6 — Supplementary Material File S1. [file NYAS-1494-70-s005.docx]

**Supporting Information**

**Primer sequences – rodent**

***ß-actin***

mActb_fo: GAAGATCTGGCACCACACCT

mActb_re: GTACATGGCTGGGGTGTTGA

rActb_fo: CACGGCATTGTCACCAACTG

rActb_re: GTACATGGCTGGGGTGTTGA

***Mchr1***

mMchr1_fo: GCATGGATCTGCAAGCCTCG

mMchr1_re: TGGTACCAAACACTGAAGGCA

rMchr1_fo: AATGCCAGCAACATCTCCGA

rMchr1_re: CTGCACCAGTGTAGCTTGGA

**Primer sequences – human**

***36B4***

h36B4_fo: CAACCCAGCTCTGGAGAAAC

h36B4_re: GTGAGGTCCTCCTTGGTGAA

***UCP1***

hUCP1_fo: GTGTGCCCAACTGTGCAATG

hUCP1_re: CCAGGATCCAAGTCGCAAGA

***MCHR1***

hMchr1_fo: CTGATGGCCCCGATAACCTC

hMchr1_re: TTCTTCACGACCGCGAAGAT

**Human biopsy material**

**Inclusion criteria:**

- Primary hyperparathyroidism requiring parathyroid surgery or elective thyroidectomy because of benign goiter, Graves’ disease, autonomous adenoma or suspected thyroid carcinoma.
- Age 20 – 55 years

**Exclusion criteria:**

- Untreated hyper- or hypothyroidism
- Current oncologic disease (other than thyroid carcinoma)
- Pregnancy
- Inflammatory bowel disease
- Active infection or use of antibiotics in the past 3 months
- Autoimmune or rheumatologic diseases that require immunosuppressive treatment
- For subjects participating in PET/CT studies additional exclusion criteria apply:
  - Use of medications which might influence BAT activity such as beta-blockers, alpha-blockers, or rilmenidin
  - Diabetes with antidiabetic medication or fasting glucose concentrations > 120 mg/dl

**Supplementary figure captions:**

**Figure S1.** Total protein staining with Ponceau S corresponding to western blots depicted in Figure 6.

**Figure S2.** Example of rat RT-qPCR melting curves.

**Figure S3**. Example of mouse RT-qPCR melting curves.

**Figure S4**. The ΔCt values (*MCHR1-36B4*) for human subcutaneous fat probes (*n =* 18) and probes of adipose tissue surrounding the carotid sheath (*n =* 6), thymus (*n =* 19), and longus colli muscle (*n =* 15).

**Figure S5.** A representative HPLC chromatogram of rat BAT 45 min after [^11^C]SNAP-7941 application (top: UV channel, bottom: radioactivity channel). The analyzed sample was spiked with the reference compound SNAP-7941.
